# Study on Mechanisms of Photon-Induced Material Removal on Silicon at Atomic and Close-to-Atomic Scale

**DOI:** 10.1007/s41871-021-00116-4

**Published:** 2021-10-27

**Authors:** Peizhi Wang, Jinshi Wang, Fengzhou Fang

**Affiliations:** 1 Centre of Micro/Nano Manufacturing Technology (MNMT-Dublin), University College Dublin, Dublin 4, Ireland; 2 State Key Laboratory of Precision Measuring Technology and Instruments, Laboratory of Micro/Nano Manufacturing Technology (MNMT), Tianjin University, Tianjin, 300072 China

**Keywords:** Atomic and close-to-atomic scale manufacturing, ACSM, Surface chlorination, Photon-induced desorption, Silicon

## Abstract

This paper presents a new approach for material removal on silicon at atomic and close-to-atomic scale assisted by photons. The corresponding mechanisms are also investigated. The proposed approach consists of two sequential steps: surface modification and photon irradiation. The back bonds of silicon atoms are first weakened by the chemisorption of chlorine and then broken by photon energy, leading to the desorption of chlorinated silicon. The mechanisms of photon-induced desorption of chlorinated silicon, i.e., SiCl_2_ and SiCl, are explained by two models: the Menzel–Gomer–Redhead (MGR) and Antoniewicz models. The desorption probability associated with the two models is numerically calculated by solving the Liouville–von Neumann equations for open quantum systems. The calculation accuracy is verified by comparison with the results in literatures in the case of the NO/Pt (111) system. The calculation method is then applied to the cases of SiCl_2_/Si and SiCl/Si systems. The results show that the value of desorption probability first increases dramatically and then saturates to a stable value within hundreds of femtoseconds after excitation. The desorption probability shows a super-linear dependence on the lifetime of excited states.

## Introduction

Manufacturing development involves three paradigms, including craft-based manufacturing, precision-controllable manufacturing, and atomic and close-to-atomic scale manufacturing (ACSM) [1, 2]. As the core competence of Manufacturing III, ACSM is directly focused on the removal/addition/migration of atoms and can be applied to the fabrication of atomic and close-to-atomic scale (ACS) features. Specifically, the material removal on silicon at ACS will play a significant role in the fabrication of chips, such as 2D transistor chips [3] and quantum chips [4, 5], in the post–Moore’s law era.

Several approaches have been proposed to achieve ACSM on silicon in the past years. One of the well-known approaches is scanning tunneling microscopy (STM)/atomic force microscopy (AFM)-based manipulation of silicon atoms using specific probes [6–8]. In addition to the direct manipulation of atoms, a near-single atom layer on the Si (100) surface can be removed by AFM probe via shear-induced mechanochemical reactions between water and silicon [9]. Although the STM/AFM-based approach has atomic precision, the low processing efficiency limits its application in industries. Another well-known approach is atomic layer deposition (ALD) [10, 11], which is used to create a conformal coating on the surface of semiconductor materials. A selected precursor is first adsorbed on the surface with a single layer, followed by a reaction step to transform the precursor into a coating layer. The sequential self-limiting steps ensure the deposition of the mono-atomic layer. However, ALD is generally applied to large-area deposition and is difficult to use in the fabrication of ACS features. Similar to ALD, thermal atomic layer etching (ALE) [12, 13] and plasma-assisted ALE [14–18] were proposed to remove silicon atoms layer by layer, in which heat or plasma was used to remove the atoms modified by precursors. Nevertheless, the spatial distributions of temperature and plasma energy are difficult to control with high precision. Therefore, the two ALE approaches fail to fabricate ACS features.

Compared with heat and plasma, photons have the advantages of high spatial resolution and highly controlled scale of energy due to their unique characteristics, such as good monochromaticity and short wavelength [19–21]. Therefore, photons are a kind of potential energy for ACSM technology in the fabrication of ACS features. Initially, photons were used to assist the etching of silicon; the mechanism of this process can be divided into thermal effects at high laser fluences and non-thermal effects at low laser fluences [22, 23]. Non-thermal effects consist of the photo-dissociation of chlorine/fluorine and carrier-enhanced chemical reaction. The reaction of photo-assisted etching is not self-limiting and therefore cannot achieve atomic or close-to-atomic precision. Several attempts were performed to apply photon irradiation to the ALE of GaAs, and a near-atomic layer precision was obtained [24, 25]. The corresponding mechanism was explained by the excitation of the GaCl-like layer to the anti-bonding state. However, a detailed calculation based on quantum mechanics was lacking, and the application to silicon was considerably difficult because the desorption energies of chlorinated silicon are notably higher than those of the chlorinated species of GaAs [26]. Photon-induced desorption experiments with photons at different wavelengths of 245, 290, and 532 nm showed that SiCl_2_ disappeared from the chlorinated silicon surface within the irradiation region, whereas SiCl remained on the surface and was stable against irradiation [27, 28]. It is believed that SiCl can be desorbed as long as a suitable wavelength of photons is selected to break the back bonds of chlorinated silicon, and this condition highly relies on the understanding of fundamental mechanisms during the corresponding process.

In this paper, the process of photon-based ACSM on silicon is first introduced. The mechanisms of photon-induced desorption in this process are studied using two desorption models. The desorption probability associated with the two models is then numerically calculated based on quantum mechanics. Finally, the calculation accuracy is verified, and the corresponding results are presented.

## Photon-based ACSM Approach

The most important aspect in the removal of target atoms without damaging bulk silicon is to ensure the self-limitation of reactions. Self-limitation implies that the reactions would stop once the target atoms are removed despite the photon irradiation of silicon. An effective way to ensure this self-limitation is a surface modification, in which the back bonds of surface atoms are weakened by specific precursors without affecting bulk silicon atoms. The weakened bonds will then be broken by photon energy, and the target atoms within the irradiation region will be subsequently removed. The underlying bulk silicon atoms will not be removed despite being exposed to photon irradiation as long as the wavelength of photons breaks the weakened back bonds but not the other bonds.

**Fig. 1:**
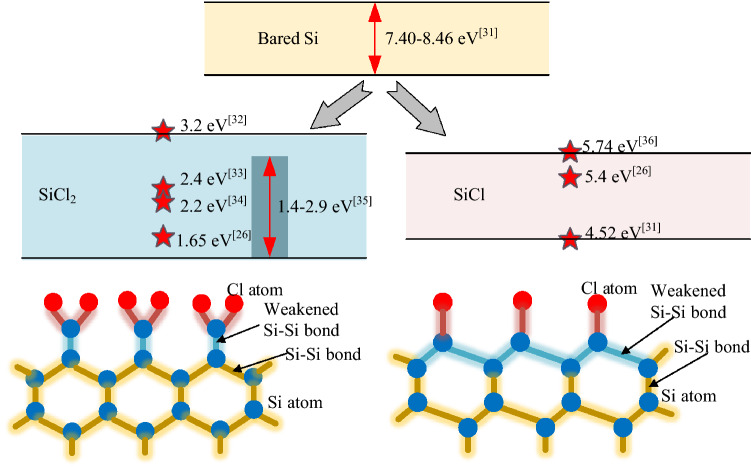
Desorption energies of bared Si, SiCl_2_, and SiCl on the silicon surface

Chlorine (Cl) can react as a precursor to modify the silicon surface by chemisorption [14, 15], in which chlorine gas spontaneously chemisorbs on the silicon surface and forms a monolayer of chlorinated silicon (mostly SiCl_2_ and SiCl) instead of SiCl_4_ [29, 30]. The chemisorbed Cl atom weakens the back bonds of silicon atoms and decreases the desorption energies of chlorinated silicon (Fig. 1). The desorption energies of SiCl_2_ and SiCl decrease from 7.40–8.46 eV to 1.4–3.2 eV and 4.52–5.74 eV, respectively, compared with bared silicon atoms [26, 31–36]. This condition provides a favorable surface for the next step, which is photon irradiation.

**Fig. 2:**
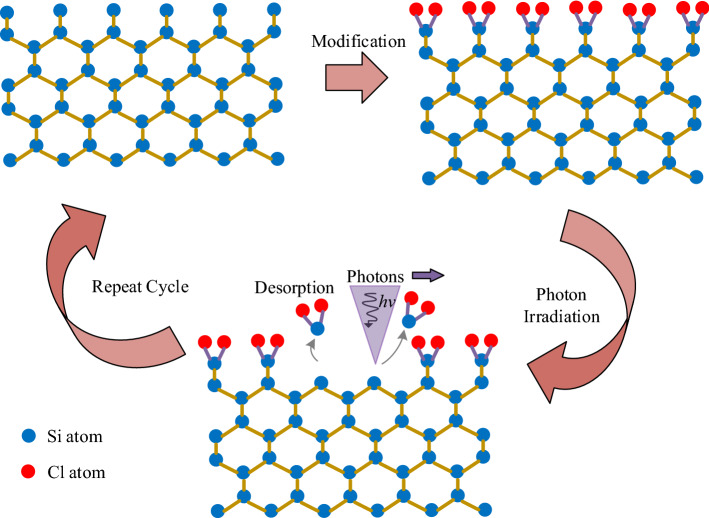
Schematic of photon-based ACSM process on silicon

Based on the above analysis, the designed photon-based ACSM approach is divided into a sequence of self-limited reactions (Fig. 2). The silicon atoms on the surface are modified by chlorine as a precursor forming a monolayer of chlorinated silicon, followed by photon irradiation to remove the modified silicon atoms. The required structures can be obtained by repeating the sequential cycle. In the irradiation step, the modified silicon atoms are removed in the form of SiCl_2_ and SiCl as a result of photon-induced desorption. The corresponding mechanisms will be discussed in the next section.

## Description of Models for Photon-Induced Desorption

Photon-induced desorption refers to the reactions in which atoms/molecules are desorbed as neutrals or ions due to electronic excitation stimulated by photons. Several models have been proposed to describe the mechanisms of photon-induced desorption; these models include the Knotek–Feibelman (KF) [37, 38], Menzel–Gomer–Redhead (MGR) [39, 40], and Antoniewicz model [41, 42]. The KF model is based on the ionization of a core level, followed by an Auger decay. The Auger process produces a two-hole state, leading to the desorption of absorbates as ions. The ionization of a core level requires photons with high energy; that is, it exceeds the threshold for the direct desorption of chlorine atoms [43]. In addition, experimental results show that the desorption products from silicon surfaces are mostly SiCl_2_ and SiCl rather than ions [27, 28]; therefore, the KF model cannot be applied to the systems of SiCl_2_/Si and SiCl/Si.

**Fig. 3:**
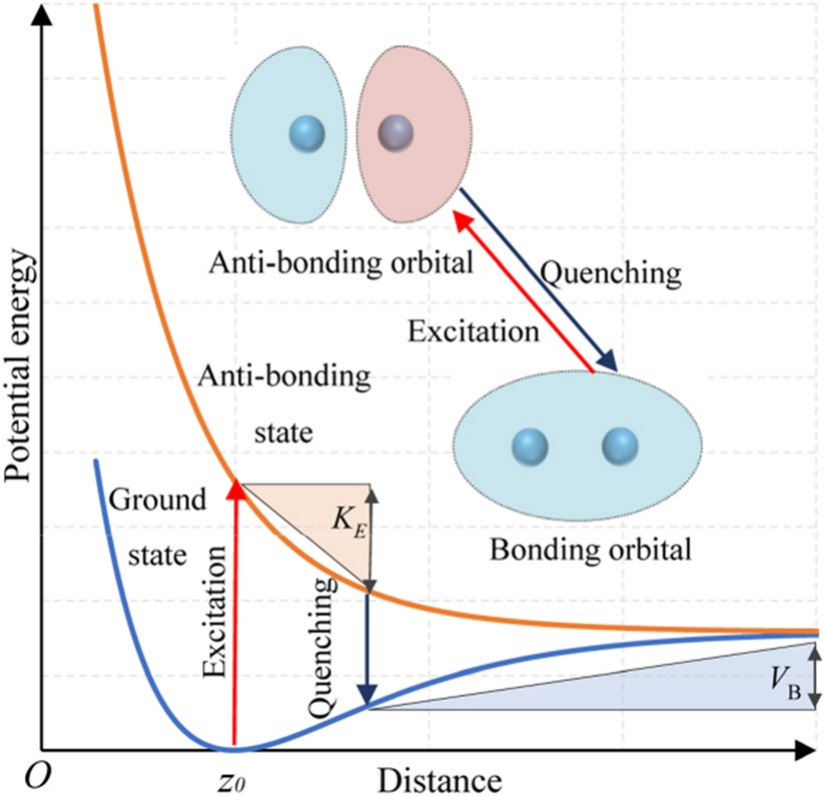
Description of MGR model for photon-induced desorption of SiCl_2_. *K*_E_ is the kinetic energy, and *V*_B_ is the potential barrier at the point of quenching

**Fig. 4:**
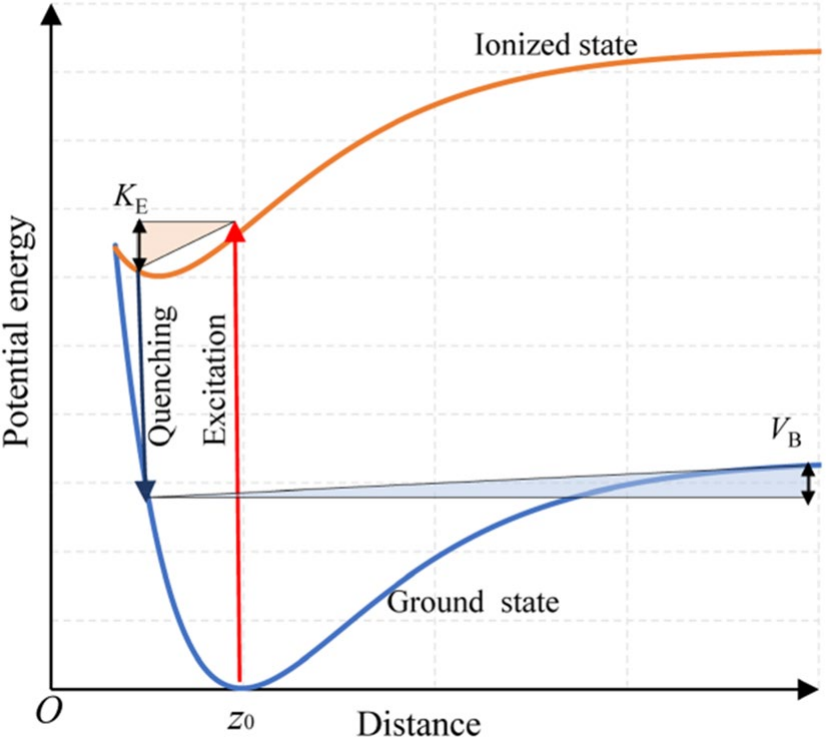
Description of Antoniewicz model for photon-induced desorption of SiCl

The mechanisms of photon-induced desorption for SiCl_2_/Si and SiCl/Si systems can be described by the MGR and Antoniewicz models, respectively (Figs. 3 and 4). For the SiCl_2_/Si system, the electrons on the back bonds of silicon are excited from the ground state to the anti-bonding state (π^*^ state for silicon [44]) after the absorption of photons. The excited adsorbate will then move away from the surface due to the associated repulsive potential effect (anti-bonding orbital shown in Fig. 3) before it quenches to the ground state. At the time of quenching, if the acquired kinetic energy *K*_E_ of the adsorbate exceeds the remaining potential barrier *V*_B_, then it will continue to move and desorb from the surface.

For the SiCl/Si system, the desorption of SiCl cannot be explained by the MGR model given that the desorption energy of SiCl is notably higher than that of SiCl_2_ (Fig. 1), and SiCl experiences difficulty in obtaining sufficient kinetic energy to overcome the potential barrier according to MGR model. Here, the Antoniewicz model can be used to explain the desorption of SiCl, in which the adsorbate is first excited to the ionized state by high-energy photons and then drops to the ground state (Fig. 4). The adsorbate will be desorbed from the surface if the acquired kinetic energy *K*_E_ exceeds the potential barrier *V*_B_. The excitation of the ionized state requires photon energy exceeding the first ionization energy of silicon, i.e., 8.15 eV [45], and this condition agrees with the results showing the stability of SiCl against the irradiation of photons at the wavelengths of 245, 290, and 532 nm [27, 28]. Photon energy should be below 17 eV to avoid the direct desorption of Cl, in which an electron from Cl 3 s orbital is excited, and an Auger-like process follows [43].

## Numerical Calculation

**Fig. 5:**
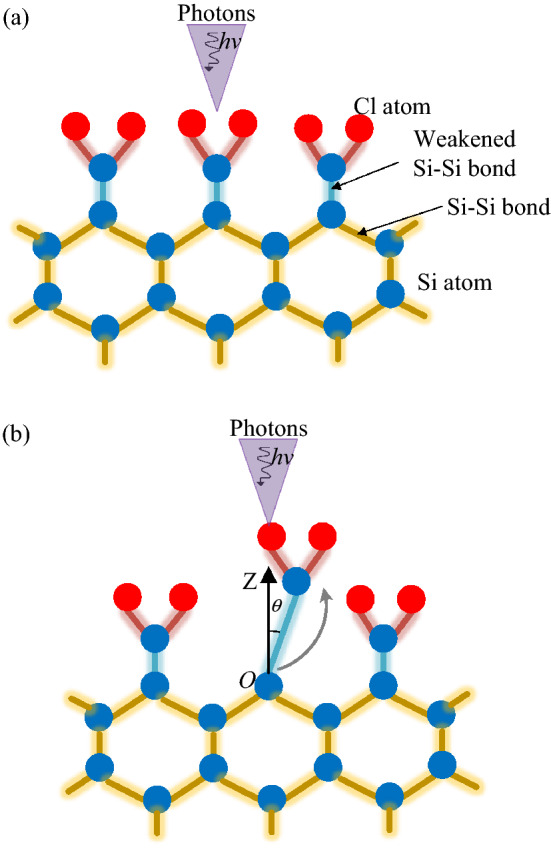
Configurations of the desorption system: **a** *t* = 0, time at the beginning of desorption; **b** *t* = *t*_1_, time after excitation

As described, the excited state in the MGR and Antoniewicz models is unstable and will quench to the ground state at a certain quenching rate. The adsorbates can desorb from the surface if the acquired kinetic energy exceeds the potential barrier at the time of quenching. Otherwise, the adsorbates will remain on the surface. In this section, the desorption probability after the excitation associated with the two models is numerically calculated based on quantum mechanics for open systems. Figure 5 shows the configurations of the desorption system. After the absorption of photons, the ground state is stimulated to the excited state, i.e., anti-bonding state or ionized state, at time *t* = 0. Then, the excited adsorbates evolve and move away from the surface along an angle *θ*. In this study, the desorption angle *θ* is set to zero because most of the adsorbates desorb along the normal direction of the surface at high coverage of chlorine [46]. In this manner, the problem can be reduced to the calculation of desorption probability in a two-state system with one dimension.

Instead of Newton mechanics, quantum mechanics for open systems is used to obtain accurate solutions. The desorption probability can be obtained by solving the Liouville–von Neumann equation [47–49]:

1 $$ \frac{{\partial \hat{\rho }}}{\partial t} = \mathcal{L}\hat{\rho } = \mathcal{L}_{\text{H}} \hat{\rho } + \mathcal{L}_{\text{D}} \hat{\rho } $$

where $\hat{\rho }$ is the density operator, *t* is the evolution time, $\mathcal{L}$ is the total Liouvillian operator, $\mathcal{L}_{\text{H}}$ and $\mathcal{L}_{\text{D}}$ are Hamiltonian Liouvillian operator and dissipative Liouvillian operator, respectively.

2 $$ \mathcal{L}_{\text{H}} \hat{\rho } = - \frac{i}{\hbar }\left[ {\hat{H},\hat{\rho }} \right] $$

3 $$ \mathcal{L}_{\text{D}} \hat{\rho } = \sum\limits_{k = 1}^{K} {\left( {\hat{C}_{k} \hat{\rho }\hat{C}_{k}^{ + } - \frac{1}{2}\left[ {\hat{C}_{k}^{ + } \hat{C}_{k} ,\hat{\rho }} \right]_{ + } } \right)} $$

where [*A*, *B*] and [*A*, *B*]_+_ denote the commutator and anti-commutator, respectively, $\hbar$ is the reduced Planck’s constant, $\hat{H}$ is the system Hamiltonian operator, $\hat{C}_{k}$ is the Lindblad operator through the dissipative channel *k*, and *k* = 1 represents the spontaneous decay from the excited state to the ground state,

4 $$ \hat{H} = \hat{H}_{e} \left| e \right\rangle \left\langle e \right| + \hat{H}_{g} \left| g \right\rangle \left\langle g \right| + \hat{V}_{eg} \left| e \right\rangle \left\langle g \right| + \hat{V}_{ge} \left| g \right\rangle \left\langle e \right| $$

5 $$ \hat{C}_{1} = \sqrt {\varGamma_{ge} } \left| g \right\rangle \left\langle e \right| $$

6 $$ \hat{\rho } = \hat{\rho }_{ee} \left| e \right\rangle \left\langle e \right| + \hat{\rho }_{gg} \left| g \right\rangle \left\langle g \right| + \hat{\rho }_{eg} \left| e \right\rangle \left\langle g \right| + \hat{\rho }_{ge} \left| g \right\rangle \left\langle e \right| $$

where $\hat{V}_{eg}$ and $\hat{V}_{ge}$ are the Hamiltonian coupling operators, $\left| g \right\rangle$ and $\left| e \right\rangle$ represent the ground and excited states, respectively, $\hat{\rho }_{mn} = \left\langle m \right|\hat{\rho }\left| n \right\rangle$ (*m*, *n* = *g*, *e*), $\varGamma_{ge}$ is the quenching rate, $\varGamma_{ge} = 1/\tau$, *τ* is the lifetime of the excited state, and $\hat{H}_{l}$ (*l* = *g*, *e*) can be represented as follows:

7 $$ \hat{H}_{l} = \hat{K} + \hat{V}_{l} \left( Z \right) = - \frac{{\hbar^{2} }}{2m}\frac{{\partial^{2} }}{{\partial Z^{2} }} + \hat{V}_{l} \left( Z \right),\;l = g,e $$

where *m* represents the molecular mass of the adsorbate, and $\hat{V}_{l}$ (*l* = *g*, *e*) represents the potential energy operators for the ground and excited states.

**Fig. 6:**
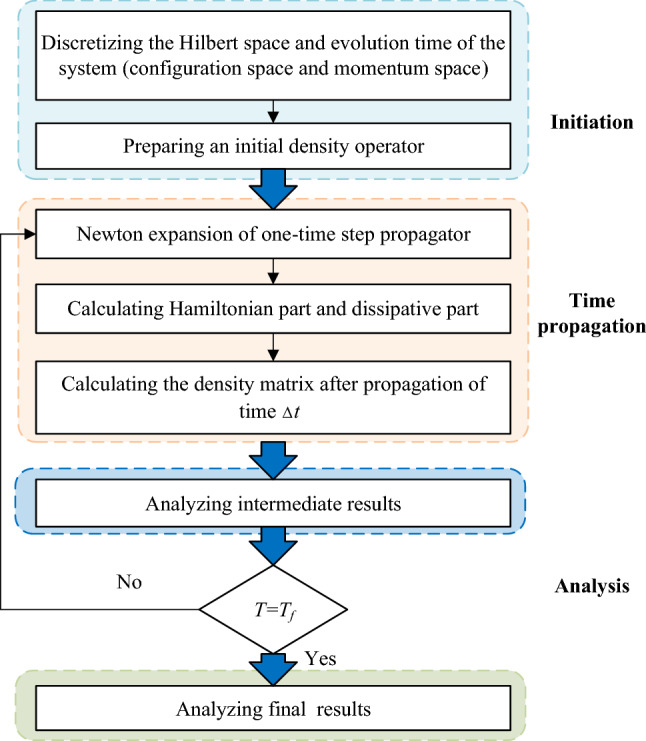
Algorithm for the numerical calculation process, which is divided into three parts: initiation, time propagation, and analysis

After obtaining the density operator $\hat{\rho }$ in accordance with the algorithm in Fig. 6, the desorption probability at any time *t* can be calculated by the following:

8 $$ P_{d} \left( t \right) = tr\left\{ {\hat{\theta }\left( {Z - Z_{d} } \right)\hat{\rho }\left( t \right)} \right\} $$

where

9 $$ \hat{\theta }\left( {Z - Z_{d} } \right) = \left\{ {\begin{array}{*{20}c} {0,{\kern 1pt} {\kern 1pt} {\kern 1pt} {\kern 1pt} {\kern 1pt} {\kern 1pt} {\kern 1pt} Z \le Z_{d} } \\ {1,{\kern 1pt} {\kern 1pt} {\kern 1pt} {\kern 1pt} {\kern 1pt} {\kern 1pt} {\kern 1pt} Z > Z_{d} } \\ \end{array} } \right. $$

where *Z*_*d*_ is the critical distance beyond which the adsorbate is considered to have been desorbed from the surface.

The algorithm in Fig. 6 includes initiation, time propagation, and analysis parts. The density operator in the coordinate representation can be calculated through the initiation and time propagation parts, as will be discussed in detail. Then, the desorption probability is calculated by Eqs. (8) and (9). Here, direct matrix manipulation and a new routine for the determination of uniformly distributed sampling points are applied to the time propagation part to improve the calculation efficiency.

In the initiation part, the Hilbert space of the system, i.e., the coordinate and momentum spaces, can be discretized by the following:

10 $$ Z_{i} = Z_{0} + \left( {i - 1} \right)\Delta Z,\;{\kern 1pt} i = 1,2, \ldots ,N $$

11 $$ {\kern 1pt} {\kern 1pt} \Delta k = \frac{2\uppi }{{\left( {N - 1} \right)\Delta Z}} $$

12 $$ {\kern 1pt} k_{i} = - \frac{\uppi }{\Delta Z} + \left( {i - 1} \right)\Delta k,\;i = 1,2, \ldots ,N $$

where *N* is the total number of discretizing points.

The initial density operator at time *t* = 0 has the following form:

13 $$ \hat{\rho }\left( 0 \right) = \hat{\sigma }\left| e \right\rangle \left\langle e \right| $$

where $\hat{\sigma }$ is a ground-state density operator and can be written as follows:

14 $$ \hat{\sigma } = \sum\limits_{v} {w_{v} } \left| {v_{g} } \right\rangle \left\langle {v_{g} } \right| $$

15 $$ w_{v} = \delta_{vv^{\prime}} $$

where $\left| {v_{g} } \right\rangle$ is the vibrational state of ground potential operator $\hat{V}_{l}$, i.e., eigenfunctions of $\hat{H}_{g}$. Here, $w_{v} = \delta_{v0}$, *δ* is the Kronecker delta function:

16 $$ \hat{H}_{g} \left| {v_{g} } \right\rangle = \varepsilon_{vg} \left| {v_{g} } \right\rangle $$

The discretizing form of $\left| {v_{g} } \right\rangle$ on coordinate representation can be obtained using the Fourier grid Hamiltonian method [50].

In the time propagation part, the density operator after one-time step (Δ*t*) of propagation can be calculated [47] by the following:

17 $$ \hat{\rho }\left( {t + \Delta t} \right) = {\text{e}}^{{\mathcal{L}\Delta t}} \hat{\rho }\left( t \right) $$

The time propagator ${\text{e}}^{{\mathcal{L}\Delta t}}$ in Eq. (17) can be expressed by Newton polynomial of order *M*:

18 $$ {\text{e}}^{{\mathcal{L}\Delta t}} \approx c_{0} \mathcal{J} + c_{1} \left( {\mathcal{L} - z_{0} \mathcal{J}} \right) + c_{2} \left( {\mathcal{L} - z_{1} \mathcal{J}} \right)\left( {\mathcal{L} - z_{0} \mathcal{J}} \right) + \cdot \cdot \cdot + c_{M} \left( {\mathcal{L} - z_{M - 1} \mathcal{J}} \right) \cdot \cdot \cdot \left( {\mathcal{L} - z_{1} \mathcal{J}} \right)\left( {\mathcal{L} - z_{0} \mathcal{J}} \right) $$

where *c*_*i*_ (*i* = 0, 1,…, *M*) are the difference coefficients, $\mathcal{J}$ is an identity operator, *z*_*i*_ is the uniformly distributed sampling point on the boundary of a complex rectangle {(0, − i*E*_max_), (0, i*E*_max_), (− *W*_max_, i*E*_max_), (− *W*_max_, − i*E*_max_)}, *W*_max_ is the maximum eigenvalue of the operator $\hat{C}_{k} \hat{C}_{k}^{ + }$, and *E*_max_ is the maximum Hamiltonian energy and can be evaluated as follows:

19 $$ E_{\max } \approx V_{\max } - V_{\min } + k_{\max }^{2} /2m $$

with $k_{\max }^{{}}$ representing the maximum momentum on the discretizing grids.

**Fig. 7:**
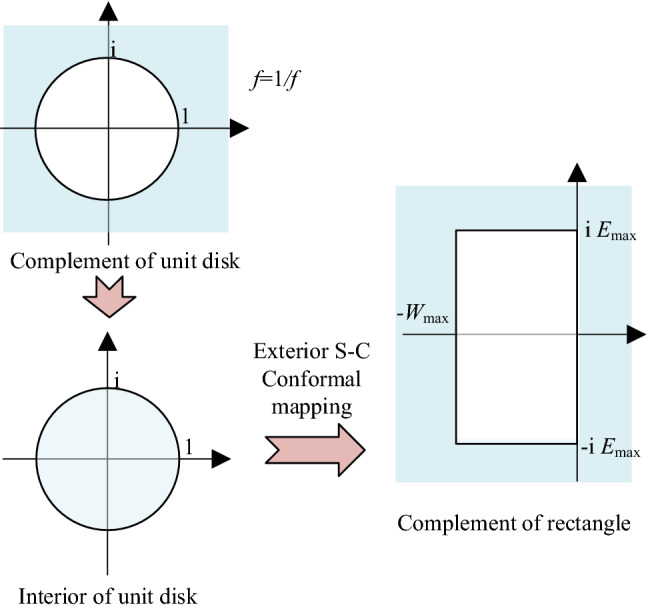
Routine for the determination of uniformly distributed sampling point *z*_*i*_

The uniformly distributed sampling point *z*_*i*_ can be obtained based on a Schwarz–Christoffel (S-C) conformal mapping method (Fig. 7). If a conformal mapping function *f*_*m*_ that can transform the complement of unit disk to the complement of a rectangle exists, then the points *f*_*m*_ (*cr*_*i*_) (*i* = 0, 1,…, *M*−1) will be the required points where *cr*_*i*_ (*i* = 0, 1,…, *M*−1) are the uniformly distributed points on the unit circle [51]. The *M*−1 points of *cr*_*i*_ are selected based on the algorithm proposed in the literature [52]. Here, the complement of the unit disk will be first transformed into the unit disk interior and then transformed again into the complement of a rectangle by exterior S-C conformal mapping [53].

Once the Newton polynomial expansion of the time propagator in Eq. (18) has been obtained, the density operator at any time can be calculated by replacing Eqs. (18) and (1) into Eq. (17). For the completion of this part, the action of $\mathcal{L}_{\text{H}}$ and $\mathcal{L}_{\text{D}}$ on $\hat{\rho }\left( t \right)$ can be directly calculated in the form of a matrix due to the powerful capability of MATLAB to deal with matrixes.

20 $$ \mathcal{L}_{{\text{H}}} \hat{\rho } = - \frac{i}{\hbar }\left[ {\left( {\begin{array}{*{20}c} {\hat{H}_{e} } & {\hat{V}_{eg} } \\ {\hat{V}_{ge} } & {\hat{H}_{g} } \\ \end{array} } \right),\left( {\begin{array}{*{20}c} {\hat{\rho }_{ee} } & {\hat{\rho }_{eg} } \\ {\hat{\rho }_{ge} } & {\hat{\rho }_{gg} } \\ \end{array} } \right)} \right] $$

21 $$ \mathcal{L}_{\text{D}} \hat{\rho } = \hat{\varGamma }_{ge} \left( {\begin{array}{*{20}c} { - \hat{\rho }_{ee} } & { - \frac{1}{2}\hat{\rho }_{eg} } \\ { - \frac{1}{2}\hat{\rho }_{ge} } & {\hat{\rho }_{ee} } \\ \end{array} } \right) $$

## Results and Discussion

In this section, the calculation accuracy of the numerical method is first verified by comparison with the results in literature in the case of the NO/Pt (111) system. Then, the desorption probabilities in the cases of SiCl_2_/Si and SiCl/Si are calculated and discussed.

### Accuracy Verification

**Fig. 8:**
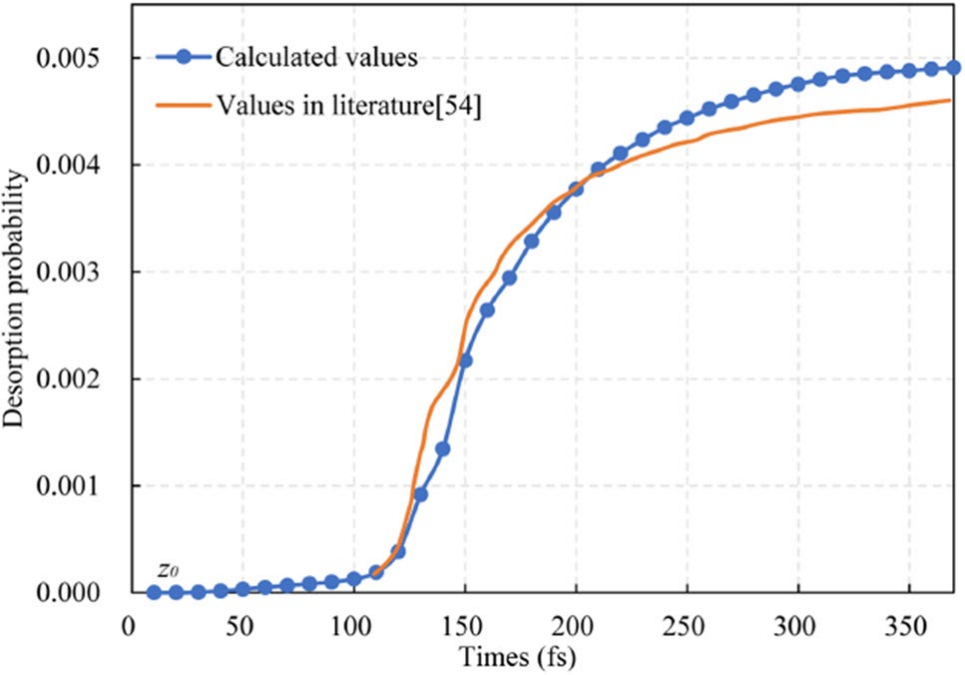
Comparison of the desorption probability between numerical calculation results and findings in literature for the NO/Pt(111) system

The reason for selecting the NO/Pt (111) system is that the system is well studied for desorption, and the related data of desorption probability can be easily obtained. Figure 8 shows a comparison of the desorption probability between the numerical calculation results and those in the literature [54]. The same potential, initiation, and propagation parameters (*τ* = 4 fs) in the literature are used for numerical calculation. The two curves have similar trends along the propagation time, and the relative error of the final values of desorption probability is 5.07%.

### Case Studies

#### SiCl_2_/Si System

The desorption probabilities in the two cases described in Sect. 3 are calculated based on the numerical calculation method. The Hamiltonian coupling operators $\hat{V}_{eg}$ and $\hat{V}_{ge}$ are neglected. The ground potentials for SiCl_2_/Si and SiCl/Si in Eq. (7) can be described by a modified Tersoff function [55]:

22 $$ V_{g} \left( Z \right) = f_{i} f_{\text{c}} \left( {Z/\cos \frac{\phi }{2}} \right)\left[ {A{\text{e}}^{{\left( { - \lambda Z/\cos \frac{\phi }{2}} \right)}} - B{\text{e}}^{{\left( { - \mu Z/\cos \frac{\phi }{2}} \right)}} } \right] $$

where *f*_*i*_ (*i* = 1, 2) is the modified coefficient describing the effect of chlorine, with *i* = 1 for SiCl_2_ and *i* = 2 for SiCl, *ϕ* is the bonding angle of Si–Si, and *f*_c_ is the cut-off function to improve the calculation efficiency.

The anti-bonding potential for SiCl_2_/Si system can be estimated as the follows [56]:

23 $$ V_{\text{e}} \left( Z \right) = f_{\text{e}} \left[ {{\text{e}}^{{\left( { - \lambda \left( {Z/\cos \frac{\phi }{2} - Z_{\text{b}} } \right)} \right)}} + {\text{e}}^{{\left( { - \mu \left( {Z/\cos \frac{\phi }{2} - Z_{\text{b}} } \right)} \right)}} } \right] $$

**Fig. 9:**
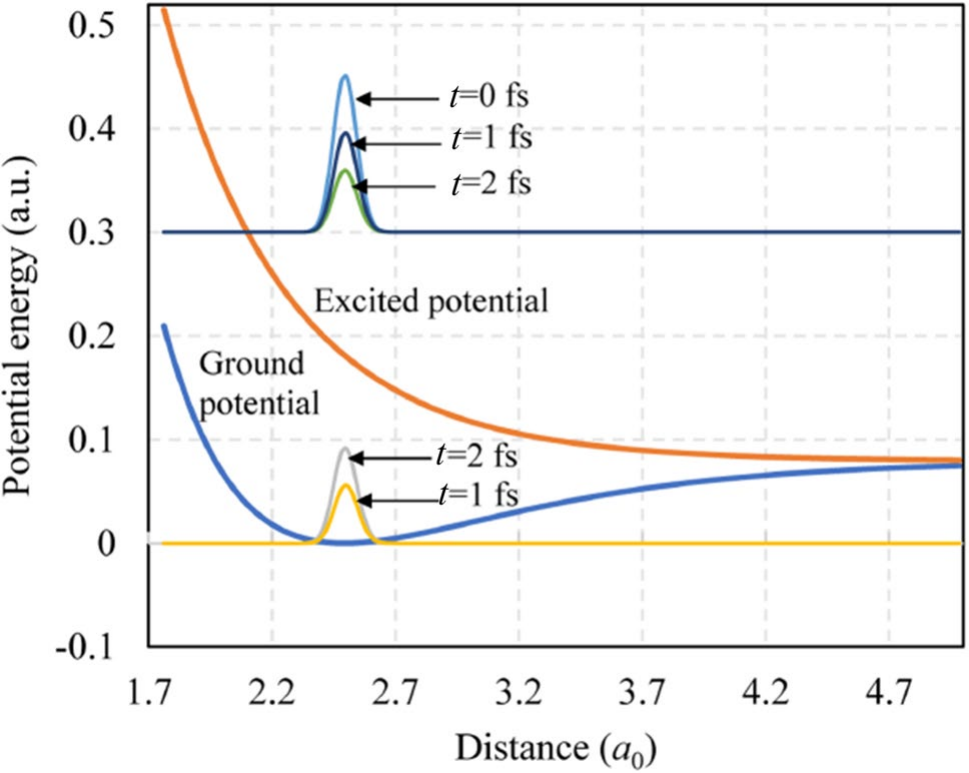
Snapshots of the density in the ground and excited states along the separation distance for the SiCl_2_/Si system. *τ* = 2 fs and *t* = 0 is the time at the beginning of desorption

Table 1 shows the potential, initiation, and propagation parameters used in the calculation. Figure 9 displays the snapshots of the density in the ground and excited states along the separation distance for the SiCl_2_/Si system. The lifetime of the excited state *τ* is assumed to be 2 fs. The ground state is excited to the anti-bonding state by Franck–Condon transition at time *t* = 0. With time propagation, the excited state gradually drops to the ground state, where the density peak of the excited state declines, and the peak of the ground state rises.

**Table 1:** Calculation parameters for the SiCl_2_/Si and SiCl/Si systems

Potential parameters [31, 35, 45, 55, 56]				
*f*_1_	0.8064	*f*_2_	1.6954	
*A*	1830.8 eV	*B*	471.2 eV	
*ϕ*	109°	*μ*	1.7322 Å^−1^	
*λ*	2.4799 Å^−1^	*f*_e_	1.32 eV	
Z_b_	2.318 Å	*Z*_e_	0.4 Å	
*V*_ion_	8.15 eV			
Initiation parameters				
*Z*_0_	1.6 *a*_0_	*N*	512	
Δ*Z*	0.018 *a*_0_			
Propagation parameters				
Δ*t*	0.5 fs	*T*_*f*_	400 fs/200 fs	
*M*	32	*τ*	1–4 fs	
*Z*_d_	6.0 *a*_0_

**Fig. 10:**
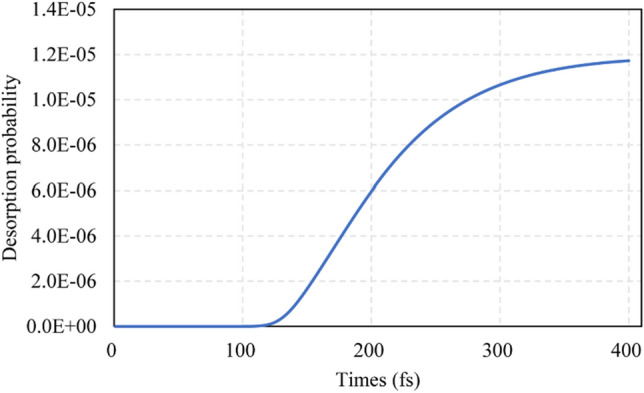
Desorption probability of SiCl_2_ as a function of propagation time for the SiCl_2_/Si system, *τ* = 2 fs

Figure 10 shows the desorption probability of SiCl_2_ as a function of propagation time. When the propagation time exceeds a critical value, the desorption probability increases dramatically and then gradually saturates to a stable value, which is the final value of desorption probability. The propagation time to reach stable values is within hundreds of femtoseconds, which means that the total reaction time for desorption is on the sub-picosecond scale.

**Fig. 11:**
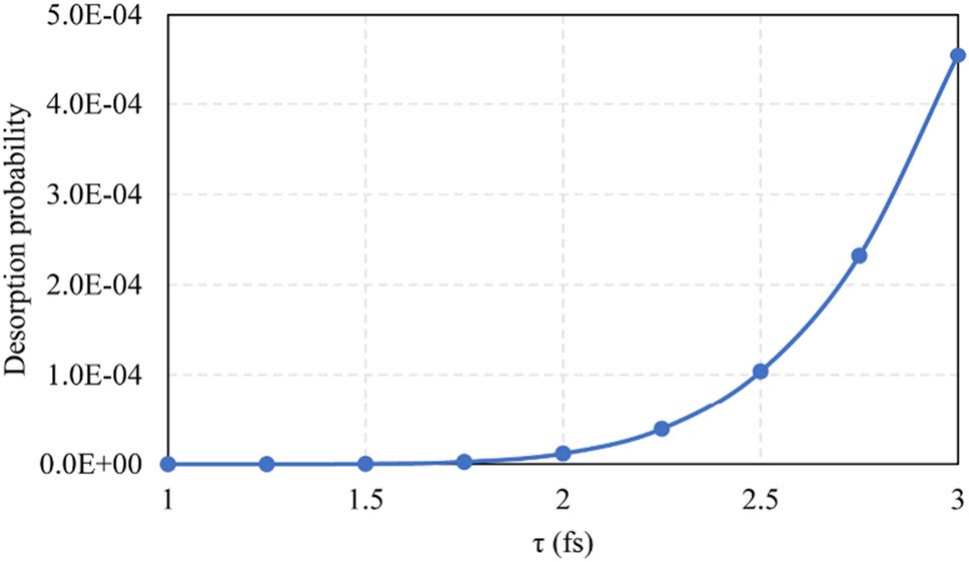
Desorption probability of SiCl_2_ as a function of excited state lifetime *τ* for the SiCl_2_/Si system

Figure 11 shows the stable value of desorption probability as a function of the lifetime of excited state *τ*. The desorption probability exhibits a super-linear dependence on *τ*. If the lifetime of the excited state can be increased, then the manufacturing efficiency of the photon-based ACSM approach will be improved significantly.

#### SiCl/Si System

The ionized-state potential for SiCl/Si system has the following form based on the ionized potential in literature [54]; the corresponding parameters are shown in Table 1.

24 $$ \begin{gathered} V_{\text{e}} \left( Z \right) = f_{2} f_{\text{c}} \left( {Z/\cos \frac{\phi }{2} + Z_{\text{e}} } \right) \cdot \hfill \\ \, \left[ {A{\text{e}}^{{\left( { - \lambda Z/\cos \frac{\phi }{2} + Z_{\text{e}} } \right)}} - B{\text{e}}^{{\left( { - \mu Z/\cos \frac{\phi }{2} + Z_{\text{e}} } \right)}} } \right] + V_{\text{ion}} \hfill \\ \end{gathered} $$

**Fig. 12:**
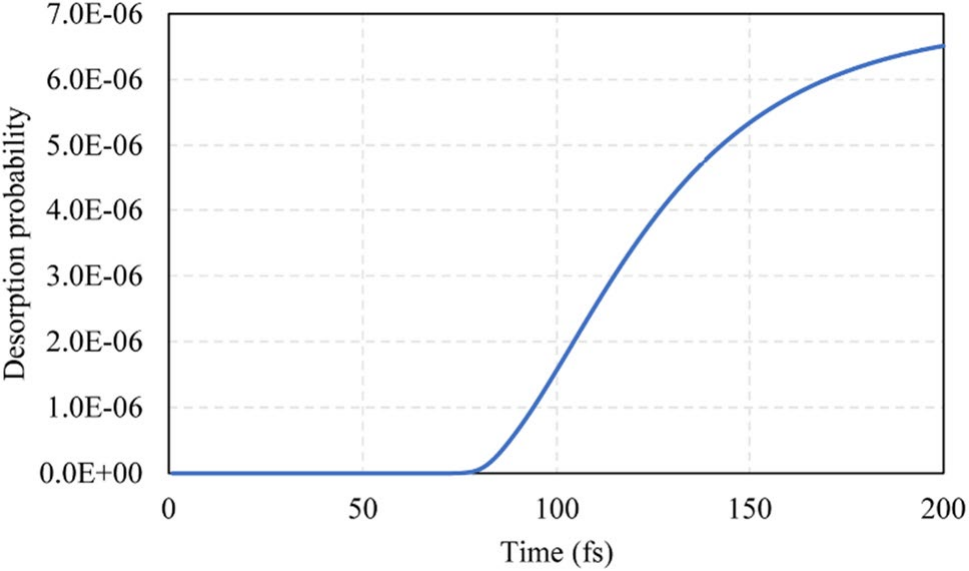
Desorption probability of SiCl as a function of propagation time for the SiCl/Si system, *τ* = 2 fs

Figure 12 shows the desorption probability of SiCl as a function of propagation time. This curve has a similar trend to the curve in Fig. 10, but the value is slightly lower. The lower value means that the desorption efficiency of SiCl is lower than that of SiCl_2_, and more efficient methods should be proposed to increase the efficiency. In addition, less time is required for SiCl to reach the saturated value than SiCl_2_ because SiCl has a smaller molecular mass.

This paper mainly investigates the mechanisms of photon-induced material removal on silicon, and experimental implementation of the proposed approach still needs to be further investigated. Although this approach is promising, the following challenges will have to be overcome, i.e., the preparation of atomic-scale flattening silicon surface, the integration of setups, and metrology. The atomic-scale flattening silicon surface is the target surface where the experiments will be carried out, and it can be obtained by wetting treatment with NH_4_F solutions. The approach requires a special-designed laser with a short wavelength, and the setups should be integrated into an ultra-high vacuum system, which is challenging. Furthermore, metrology requires atomic-scale precision and online measurements. Therefore, STM/AFM may need to be integrated into the system.

## Conclusions

A new approach for the photon-induced material removal on silicon at ACS is developed, and the corresponding mechanisms are investigated. The main conclusions from this study can be drawn as follows.

1. A new approach for ACSM on silicon is proposed by using chlorine to modify the silicon atoms on the surface and then using photon irradiation to desorb the modified silicon atoms in the form of SiCl_2_/SiCl.
2. Mechanisms of the photon-induced desorption of SiCl_2_ and SiCl from the silicon surface are studied by the MGR model, corresponding to the anti-bonding state, and the Antoniewicz model, corresponding to the ionized state. The desorption probability associated with the two models is numerically calculated based on quantum mechanics. The calculation accuracy is verified by comparing with the results in literature in the case of NO/Pt (111) system with the relative error of 5.07%.
3. The values of desorption probability first increase dramatically and then saturate to stable values with propagation time within hundreds of femtoseconds. The results also show the super-linear dependence of desorption probability on the lifetime of the excited state, which indicates that the increase in this lifetime will help in increasing the manufacturing efficiency significantly.
